# Overexpression of Homer1a in the basal and lateral amygdala impairs fear conditioning and induces an autism-like social impairment

**DOI:** 10.1186/s13229-016-0077-9

**Published:** 2016-02-29

**Authors:** Anwesha Banerjee, Jonathan A. Luong, Anthony Ho, Aeshah O. Saib, Jonathan E. Ploski

**Affiliations:** Department of Cell Biology, Emory University, 615 Michael St. WBRB #415, Atlanta, GA 30322 USA; School of Behavioral and Brain Sciences, University of Texas at Dallas, 800 West Campbell road, Richardson, TX 75080 USA

**Keywords:** Autism, Learning, Memory, Valproic acid, Amygdala, Emotion, Pavlovian fear conditioning, Homer1a

## Abstract

**Background:**

Autism spectrum disorders (ASDs) represent a heterogeneous group of disorders with a wide range of behavioral impairments including social and communication deficits. Apart from these core symptoms, a significant number of ASD individuals display higher levels of anxiety, and some studies indicate that a subset of ASD individuals have a reduced ability to be fear conditioned. Deciphering the molecular basis of ASD has been considerably challenging and it currently remains poorly understood. In this study we examined the molecular basis of autism-like impairments in an environmentally induced animal model of ASD, where pregnant rats are exposed to the known teratogen, valproic acid (VPA), on day 12.5 of gestation and the subsequent progeny exhibit ASD-like symptoms. We focused our analysis on the basal and lateral nucleus of the amygdala (BLA), a region of the brain found to be associated with ASD pathology.

**Methods:**

We performed whole genome gene expression analysis on the BLA using DNA microarrays to examine differences in gene expression within the amygdala of VPA-exposed animals. We validated one VPA-dysregulated candidate gene (*Homer1a*) using both quantitative PCR (qRT-PCR) and western blot. Finally, we overexpressed Homer1a within the basal and lateral amygdala of naïve animals utilizing adeno-associated viruses (AAV) and subsequently examined these animals in a battery of behavioral tests associated with ASD, including auditory fear conditioning, social interaction and open field.

**Results:**

Our microarray data indicated that *Homer1a* was one of the genes which exhibited a significant upregulation within the amygdala. We observed an increase in *Homer1a* messenger RNA (mRNA) and protein in multiple cohorts of VPA-exposed animals indicating that dysregulation of Homer1a levels might underlie some of the symptoms exhibited by VPA-exposed animals. To test this hypothesis, we overexpressed Homer1a within BLA neurons utilizing a viral-mediated approach and found that overexpression of Homer1a impaired auditory fear conditioning and reduced social interaction, while having no influence on open-field behavior.

**Conclusions:**

This study indicates that dysregulation of amygdala Homer1a might contribute to some autism-like symptoms induced by VPA exposure. These findings are interesting in part because Homer1a influences the functioning of Shank3, metabotropic glutamate receptors (mGluR5), and Homer1, and these proteins have previously been associated with ASD, indicating that these differing models of ASD may have a similar molecular basis.

**Electronic supplementary material:**

The online version of this article (doi:10.1186/s13229-016-0077-9) contains supplementary material, which is available to authorized users.

## Background

Autism spectrum disorders (ASD) represent an etiologically heterogeneous group of disorders that are believed to be caused by a myriad of genetic and environmental factors. ASD patients typically exhibit symptoms of repetitive behavior, impaired social interactions and deficits in social communication. These patients also frequently exhibit intellectual disability, epilepsy, attention deficit hyperactivity disorder (ADHD), sleep disturbances [1–3], and anxiety [4, 5]. Additionally, some ASD patients may exhibit a reduced ability to be fear conditioned [6].

It is difficult to trace the pathology of a heterogeneous disorder such as ASD to a single brain region. However, the presence of significant social and emotional abnormalities among the human ASD population indicates atypical amygdala function including the basolateral complex which plays an important role in processing emotional and social cues [7–13]. Collectively, more research is needed to further elucidate the underlying molecular and cellular basis of amygdala dysfunction in ASD.

Twin and family studies have indicated that there is a strong genetic basis for ASD [14–18]. The genetics of ASD is highly complex, involving multiple genes with a high degree of genetic variation [19, 20]. Notably, a recent study found that siblings with ASD often possess very different genomic mutations, indicating that even within a single family, the cause of ASD can be heterogeneous [21]. Although the genetic basis of ASD is well documented, the recent increase in clinical cases of idiopathic ASD indicates that environmental risk factors might also have an important contribution, either by causing new mutations or by increasing the risk for ASD in a genetically predisposed individual [22–24].

Studies utilizing animal models of ASD have significantly increased over the past decade, allowing researchers to gain a better understanding of the neurobiology of ASD. One such animal model is the valproic acid (VPA) model of autism [25–27]. This model is based on the discovery that administration of the anticonvulsant drug VPA during the first trimester of pregnancy increases the likelihood of having children with ASD and intellectual disability [22–24, 28, 29]. In this animal model, pregnant dams are administered a single dose of VPA on or around embryonic day 12.5 of gestation, during the time of neural tube closure. The resultant progeny display anatomical and behavioral abnormalities similar to human ASD [26, 30–32], including deficits in social interaction, increased levels of anxiety, and abnormal fear learning [33–36]. Several independent groups have speculated that synaptic deficits may contribute an important role in the causal mechanism of ASD. Synaptic abnormalities have been observed in fragile X [37, 38], Rett syndrome [39], Angelman syndrome [40], and tuberous sclerosis complex [41–43]. Proteins required for normal synaptic functioning have also been implicated in ASD such as Shank, MeCP2, Reelin, and Neuroligin. [43–47]. These proteins contribute an important role to synaptic plasticity and learning and memory.

In the current study, we aimed to understand the molecular basis of amygdala dysfunction, in animals exposed to VPA in utero. We performed genome-wide gene expression profiling on amygdala tissue obtained from adult rats that were exposed to VPA in utero. We identified Homer1a, which is a dominant negative regulator of the critical synaptic scaffolding protein Homer1, to be significantly upregulated in the basal and lateral amygdala (BLA) of VPA-exposed animals. Homer1a is especially interesting since it interacts directly with important synaptic proteins such as metabotropic glutamate receptors (mGluR5) and Shank, which have previously been associated with ASD [48–54]. Additionally, Homer1a contributes a critical role in plasticity and fear learning [55–58] indicating that its dysregulation could underlie fear learning abnormalities observed in VPA-exposed animals. Therefore, we hypothesized that overexpression of Homer1a in the BLA could impair amygdala-dependent phenomena such as fear learning or social interaction behavior [11, 59–63]. To test our hypothesis, we used a viral-mediated approach and overexpressed Homer1a in the BLA of naive animals. We found that overexpression of Homer1a in the BLA impaired fear learning and reduced social interaction in the animals but had no influence on locomotor behavior or anxiety as measured by the open-field test. Our findings are intriguing in part because the Homer1 gene has been previously associated with the human ASD population [64] thus underscoring that an environmentally induced animal model of ASD could provide novel avenues for elucidating the neurobiological mechanisms of ASD.

## Methods

### Subjects

#### VPA animals

To obtain progeny exposed to either VPA or saline in utero, rats were mated overnight and pregnancy was determined by the presence of a vaginal plug (E1). Dams were given a single intraperitoneal injection of 600 mg/kg of VPA in 0.9 % saline or saline alone (control) on day E12.5 of pregnancy as previously described [30, 65]. All dams were housed individually and left undisturbed until they gave birth. The offspring were weaned on postnatal day (PD) 21, and animals of either sex were housed separately. A total of 13 saline litters and 19 VPA litters from 4 different cohorts were used for the molecular experiments. Animals exposed to VPA/saline were sacrificed for molecular experiments at approximately PD 90, and these animals were sacrificed in a counterbalanced fashion across the groups. *Naïve* adult male Sprague Dawley rats (*Harlan*) weighing 300–400 g were used for the experiments where adeno-associated viruses (AAV) were infused into the BLA. All animals were housed individually and maintained on a 12 h light/dark cycle. Food and water were provided ad libitum throughout the experiments. Animal use procedures were in accordance with the National Institutes of Health Guide for the Care and Use of Laboratory Animals and were approved by the University of Texas at Dallas Animal Care and Use Committee.

#### DNA microarray and analysis

Animals were lightly sedated by exposing them to CO_2_ for ~1 min, followed by immediate decapitation; the brains were rapidly dissected and immediately frozen with powdered dry ice and stored at −80 °C until further processing. Unilateral amygdala punches were obtained with a 1-mm punch tool (Fine Science Tools), and total RNA was isolated using RNAqueous Micro kit (Ambion). The resultant RNA was purified via precipitation using Pellet Paint NF (Novagen) to remove potential inhibitors of reverse transcription followed by RNA amplification and biotin labeling using the MessageAmp II-Biotin Enhanced Single Round cRNA Amplification Kit (Ambion). DNA microarray hybridization was performed at the University of Texas at Southwestern Medical Center Genomics and Microarray Core Facility. Eight cRNA samples (*n* = 4 for VPA, *n* = 4 for saline) were hybridized to Affymetrix GeneChip Rat Genome 230 2.0 Arrays containing 31,000 probes for genome scale gene expression analysis. Gene lists were created based on the criteria that the gene must exhibit an average fold difference of 1.5-fold or greater in pairwise comparisons between VPA and saline amygdala samples with a non-corrected, *t* test *p* value of *p* < 0.05. The MAQC Consortium has reported that this approach can be successful in identifying reproducible gene lists [66].

#### Laser microdissection, RNA purification, cDNA synthesis, and qRT-PCR

Laser microdissection of basal and lateral amygdala (BLA) followed by RNA purification, complementary DNA (cDNA) synthesis and quantitative PCR (qRT-PCR) were performed as described previously [67]. Briefly, hemisected 8–10 μm coronal sections containing the amygdala (−2.3 to −3.3 mm with respect to bregma) were mounted on MMI laser microdissection (LMD) slides (product #50102). Prior to LMD, the slides were dehydrated and the BLA nuclei were laser microdissected using a SmartCut Laser Microdissection System configured on an Olympus CKX41 inverted microscope. Each microdissected BLA/slice was deposited in 25 μL of cell lysis buffer (RNAqueous-Micro Kit; Ambion). This was repeated for ~6–7 slices per animal per side, and the resultant 25 μl aliquots were frozen at −80 °C until further processing. The samples were thawed and pooled, and the RNA was isolated according the manufacturer’s instructions using the RNAqueous-Micro Kit. The resultant RNA was purified via precipitation using Pellet Paint NF (Novagen). RNA was converted to cDNA for each sample in a 20 μL reaction using 250 nM of oligodT (Invitrogen), 1 μL reverse transcriptase (Invitrogen), and 1 μL SUPERase In (Ambion). The cDNA was purified using Pellet Paint NF (Novagen) and re-suspended in 100 μL of nuclease free water. One microliter of cDNA was used per 20 μL Taqman PCR assay (Applied Biosystems). One microliter of a 20X Taqman custom Primer/Probe for Homer1a or Homer1 (Rn00581785_m1 FAM) (Invitrogen) was used per reaction. The primer sequence for Homer 1a is FP 5′-CTGCTCCAAAGGAAAGCCTTGC-3′ and RP 5′-AAACAACCTTCAATGCTGACGG-3′. The probe sequence is 5′-[FAM] CGTCCTCTGTGGCACCTCTGTGGGC [TAMRA]-3′ [68]. Samples were prepared and loaded into a 96-well plate in triplicate and relative gene concentrations were normalized and quantitated against glyceraldehyde-3-phosphate dehydrogenase (GAPDH) (Rn01775763_g1 VIC; Invitrogen), levels using CFX96 real-time PCR system (BioRad) using the standard cycling parameters specified by Applied Biosystems. The data were analyzed using a two-tailed *t* test. Differences were considered significant if *p* < 0.05. For the most accurate representation of qRT-PCR experimental data variance, all qRT-PCR data are represented as the average threshold cycle (Ct) difference values for each group after normalization to GAPDH, with the error bars representing the standard error of the mean for each group. Average fold change values result from the transformation of the raw qRT-PCR data using the equation: 2^(average Ct difference value)^ = average fold change.

#### AAV production, purification, and titering

AAV2 genome plasmids which are designed to express either Homer1a or GFP were generously provided by Dr. Matthias Klugmann [69]. Specifically, the expression cassettes contain a 1.1-kb CBA promoter composed of 266 bp of the cytomegalovirus immediate early enhancer and 410 bp sequence containing exon 1 of CBA, a hybrid CBA/rabbit β-globin intron and the 5′ end of a β-globin exon which control the expression of an HA-tagged Homer1a transgene or eGFP transgene [70]. The AAV2 viral plasmids used for the second set of viral experiments were generously provided by Dr. Martin Schwarz [58]. They are designed to express either venus tagged Homer1a (H1aV) or a similar control venus tagged Homer1a mutant (H1aV(W24A)) containing a point mutation (W24A) in the EVH1 domain of H1aV, which abolishes the ability of Homer1a to interact with postsynaptic protein partners. These plasmids have an AAV backbone containing the 480 bp human synapsin core promoter. All viral plasmids within this study contain a woodchuck posttranscriptional regulatory element (WPRE) and the bovine growth hormone polyA sequence. AAV viruses used within this study were pseudotyped to create AAV2/DJ8 viruses, and these were produced and purified using a triple transfection method in 293FT cells and purified on an iodixanol step gradient as described previously [71]. Viral titers were determined using a quantitative-PCR-based titering method using a CFX96 real-time PCR system (Bio-Rad) using the standard cycling parameters specified by Applied Biosystems with custom Taqman primer/probes directed to the WPRE sequence within the viral genome. The primer sequences for WPRE are FP 5′-CCGTTGTCAGGCAACGTG-3′ and RP 5′-AGCTGACAGGTGGTGGCAAT-3′. The WPRE probe sequence was 5′-[FAM]-TGCTGACGCAACCCCCACTGGT-[TAMRA]-3′. Final viral titers were computed based on a standard curve and reported as genome copies (GC/ml) as previously described [71].

#### AAV infusions

Twenty-eight gauge custom-made infusion cannula (C315G, Plastics One) were inserted into polyethylene tubing (I.D. 0.015 in., O.D. 0.043 in., wall thickness 0.0140 in.) (A-M systems, Inc.) which were ~20 in. long, and then, these lines were backfilled with sesame oil and attached to 2 μL, 23-gauge (88500) stainless steel Hamilton syringes (Hamilton Company). Under a mixture of ketamine (100 mg/kg) and xylazine (10.0 mg/kg) anesthesia, rats were stereotaxically implanted bilaterally with the 28-gauge infusion cannula (described above) targeting the BLA [AP −2.9, ML ±5.0, DV −8.6]. Viruses were bilaterally infused for 15 min at a rate of 0.09 uL/min. A total of 1 uL of virus consisting of a viral titer of ~1.5E13 GC/mL was infused per side. For animals that received the HA-Homer1a virus, the HA-Homer1a virus was mixed with a very small amount of the GFP control virus (6.5E8 viral particles) and then infused. This was to allow for visualization of the viral transduction within the BLA, to confirm the virus was indeed correctly placed and virus was successfully infused into the BLA. The total number of viral particles was kept constant between both of the GFP control group and the HA-Homer1A group. Following infusions, the infusers were left in for ten additional minutes to allow diffusion of the virus away from the cannula after which they were withdrawn, and the incision was closed using 9 mm wound clips (Mikron Precision, Inc.). The animals were allowed to heal for a week, and the wound clips were removed after a week using a wound clip remover (Autoclip, Mikron Precision, Inc). Three weeks post infusion, the animals were tested on a battery of behavioral tests. Each behaviorally tested animal was handled for 2 days prior to behavior tests. After the completion of behavioral tests, the rats were sacrificed in a counterbalanced fashion across the groups by anesthetizing with an overdose of chloral hydrate (250 mg/kg) and perfused with phosphate buffered saline (1X phosphate buffer, 150 mM NaCl) and 10 % buffered formalin. The brains were fixed in 10 % formalin for 4–5 h followed by cryoprotection in 30 % sucrose in 1X PBS. Only animals which possessed correctly placed viral transduction for each experiment were included in the analysis.

#### Western blot

Rats were decapitated after sedation in CO_2_, and brains were frozen at −80 °C until further processing. Punches containing the BLA were obtained with a 1-mm punch tool (Fine Science Tools) from 200-μm-thick sections taken on a cryostat. Punches were manually dounced homogenized in 200 μL of ice-cold lysis buffer [10 mM Tris-HCl, pH 7.5, 1 mM EDTA, 2.5 mM sodium pyrophosphate, 1 % NP-40, 0.5 % sodium deoxycholate, 1 % SDS, 1 mM DTT, protease inhibitor cocktail (Roche)]. Protein concentrations were assessed and normalized across homogenates using a BCA kit (Thermo scientific). Sample buffer was added to the homogenates, and the samples were boiled for 5 min. Homogenates were electrophoresed on 5–15 % Tris-HCl gels (Bio-rad) and transferred overnight to Immobilon-P (Millipore) at 4 °C. Western blots were blocked in TTBS buffer (50 mM Tris-HCl, pH 7.5, 150 mM NaCl, and 0.05 % Tween 20) with 5 % nonfat dry milk (American Bioanalytical) for 1 h and then incubated with the following primary antibodies: Homer1a antibody (1:1000; cat # 160013, Synaptic Systems), anti-HA antibody (1:1000; cat# MMS-101P, Covance), and anti-GAPDH (1:5000; cat# ab9483, Abcam) overnight at 4 °C on a shaker. Blots were then incubated with anti-rabbit or anti-mouse antibodies conjugated to horseradish peroxidase (Cell Signaling) for 1 h at room temperature and developed using West Pico chemiluminescent substrate (Pierce Laboratories). Densitometry was conducted using NIH ImageJ software. To control for inconsistencies in loading, optical densities were normalized to GAPDH proteins.

#### Auditory fear conditioning

Animals were fear conditioned 21 days post viral infusion. A Coulbourn Instruments fear conditioning system with computer-controlled shockers, USB cameras for video monitoring/video capture, and FreezeFrame Software (Actimetrics) for unbiased behavioral analysis was used to auditory fear condition rats and to test for conditioned fear responses.

#### Training

Rats were auditory fear conditioned with a single trial consisting of a 180-s acclimation period (pre-shock period) followed by the presentation of a 30-s, 5-kHz, 75-dB tone that co-terminated with a 1-s, 1.5-mA foot shock. Animals remained in the training chamber for an additional minute following the delivery of the foot shock, and subsequently, the animals were placed back into their home cages.

#### STM

Animals were tested for retention of Short-term memory (STM) 3 h post fear conditioning in a novel context which had distinct tactile, olfactory, and visual cues compared to the auditory fear conditioning training chamber. STM testing consisted of a 1-min acclimation period followed by the presentation of (30 s, 5 kHz, 75 dB) tones with an inter-trial interval of 2 min. Following the last tone, animals remained in the box for an additional 1 min and were subsequently returned to their home cages.

#### LTM

Animals were tested for long-term memory (LTM) 24 h post auditory fear training in the same manner as STM was. All trials were recorded using Freeze frame software. The absence of any movement excluding respiration was recorded as a freezing response, which was calculated by the automated Freeze frame software.

#### Anxiety/locomotory behavior

General anxiety/innate fear and locomotor behavior were examined in an open field. The open-field apparatus consisted of a wooden square (1.2 m × 1.2 m) box. The periphery of the box was designated as the outer 0.30 m region of the box, and the center zone was designated the 0.6 m × 0.6 m square region at the center of the box. Each animal was placed in a corner of the box and allowed to freely explore the field for 10 min in low light (~100 lux) while being video recorded using a USB camera. After the session, the animal was removed from the apparatus and returned to the home cage and the open-field apparatus was cleaned with 10 % ethanol and allowed to dry completely between trials. Total entries to the center of the open field, total time spent at the center, total distance traveled, and mean speed for each animal were calculated by the automated behavioral tracking system, ANYmaze (Stoelting). The total distance traveled for each animal was used to compare overall locomotory behavior between the groups. Differences in open-field center time between the groups were used to determine if there were differences in anxiety/innate fear.

#### Social interaction test

Social interaction between rats was measured using a protocol similar to one described previously [65]. On day 1, the subject rat (Homer1a or control animal) was habituated to the empty social interaction apparatus (61 cm × 61 cm gray box) for 5 min. On day 2, a wired enclosure was placed in the corner of the social interaction box and a novel test rat which had no previous exposure to the subject rat was placed inside this enclosure. The subject rat was then introduced into the social interaction box and allowed to interact with the novel test rat for 10 min. The presence of the test rat in the wired enclosure allowed the measurement of social behavior initiated by the subject rat only. The region surrounding the novel test rat chamber was marked as the social interaction zone, (5-cm-wide region surrounding the outside of the wired enclosure). The social interaction apparatus was wiped down with 10 % ethanol between animals to remove olfactory cues from previous trials. The amount of time spent in the social interaction zone, sniffing, and exploring the test rat was recorded by automated ANYmaze software and considered as a measure of social interaction behavior. Mean distance traveled in the apparatus measured the overall locomotory behavior of the animals.

### Statistical analysis

All statistics were performed using SPSS 12.0 or StatView. Results were expressed as mean ± SEM in the text and figures. Data were analyzed for normality using Shapiro-Wilk test. Nonparametric statistical tests were used in experiments with small sample sizes (*n* < 10) [72, 73]. Equivalent parametric tests for the same were also performed and reported (Additional file 1). Within group, comparisons for repeated measure were performed using Friedman tests followed by Mann-Whitney *U* tests for between group comparisons as described previously [74]. A Student’s *t* test was used to compare means between groups where appropriate. A paired Student’s *t* test was used to compare means within group. Differences with *p* < 0.05 were considered statistically significant.

## Results

### In utero VPA exposure dysregulates gene expression within the adult rat amygdala

**Fig. 1:**
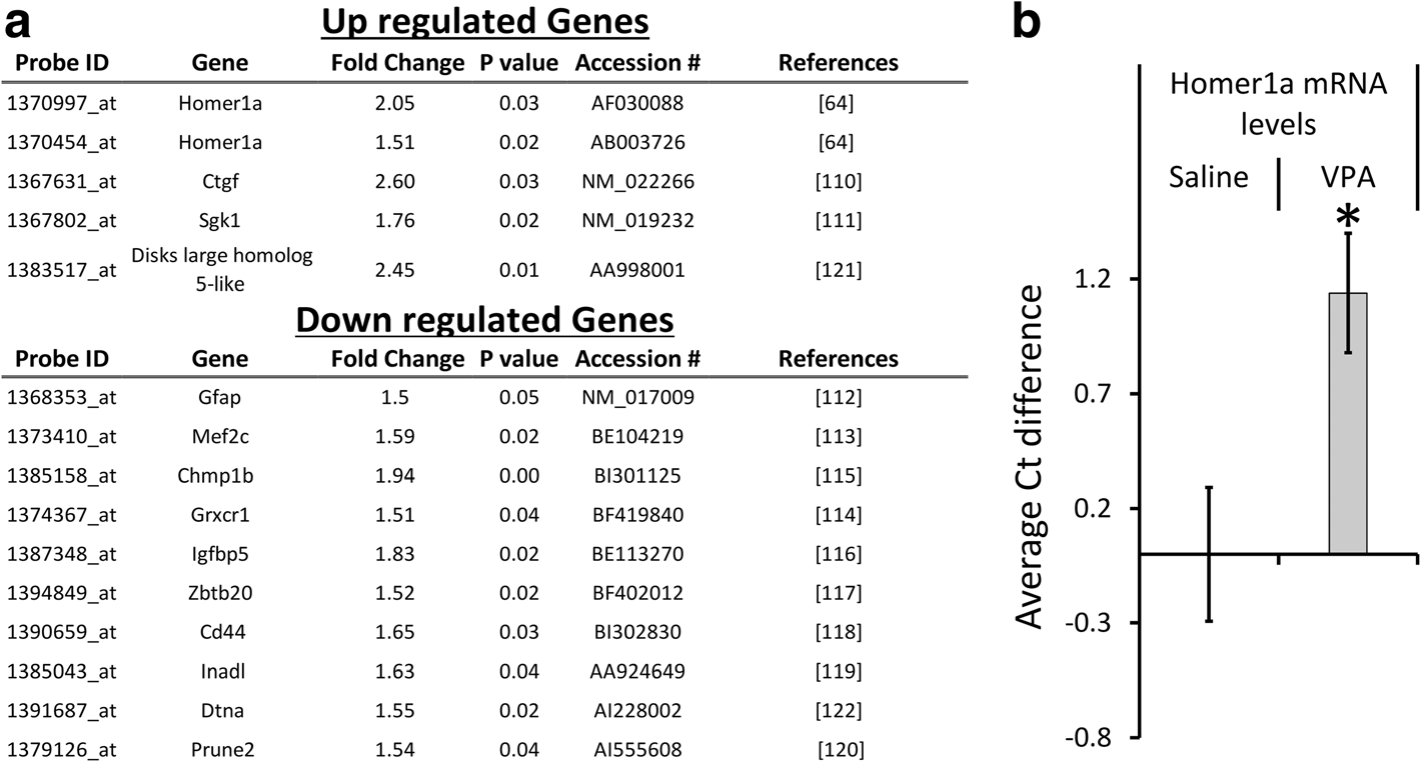
Whole genome microarray indicates Homer1a is upregulated in the BLA of VPA-exposed animals. **a** The table depicts the list of genes dysregulated in the basolateral amygdala (BLA) of VPA-exposed animals compared to saline-exposed animals, which also have been previously associated with ASD (*p* < 0.05) [64, 110–122]. **b** Upregulation of Homer1a was validated using qRT-PCR with *Homer1a*-specific probes using the same aRNA samples that were examined via DNA microarray. *Bars* represent the mean ± standard error of the mean (SEM) (*p < 0.05)

To determine if exposure to VPA in utero influences gene expression in the BLA, we performed whole genome, gene expression profiling using Affymetrix DNA microarray gene chips. Total RNA was purified from BLA tissue (saline = 4, VPA = 4) and subjected to RNA amplification and biotin labeling followed by genome microarray hybridization and analysis. A total of 59 genes were differentially expressed (>1.5-fold change; *p* < 0.05) in the BLA of VPA-exposed animals compared to saline animals (Additional file 2). Interestingly, 14 of the 59 genes identified by the microarray screen (i.e., 23.7 %) have been previously associated with human ASD and these genes are listed in Fig. 1a. *Homer1a* was identified as one of the genes significantly upregulated in the BLA of VPA-exposed animals. Two independent probe sets (Probe Set ID: 1370997_at, 1370454_at) on the DNA microarray indicated that *Homer1a* was upregulated. Because Homer1a partakes in mGluR5 signaling and regulates interactions of long forms of Homer1 with Shank, and all three of these proteins have been previously associated with ASD, we focused our attention on Homer1a. To begin our validation to determine if *Homer1a* is indeed upregulated within the amygdala due to VPA exposure, we performed qRT-PCR on the amplified RNA (aRNA) samples used for DNA microarray analysis. An average Ct (cycle threshold) difference of 1.14 ± 0.2 was found between the VPA- and saline-exposed groups, which corresponds to a 2.2 average fold upregulation [*t*_(7)_ = 2.82, *p* = 0.02] of *Homer1a* messenger RNA (mRNA) in VPA animals, and these values are consistent with the microarray data (Fig. 1b).

### Homer1a mRNA and protein are upregulated within the adult BLA from VPA-exposed animals

**Fig. 2:**
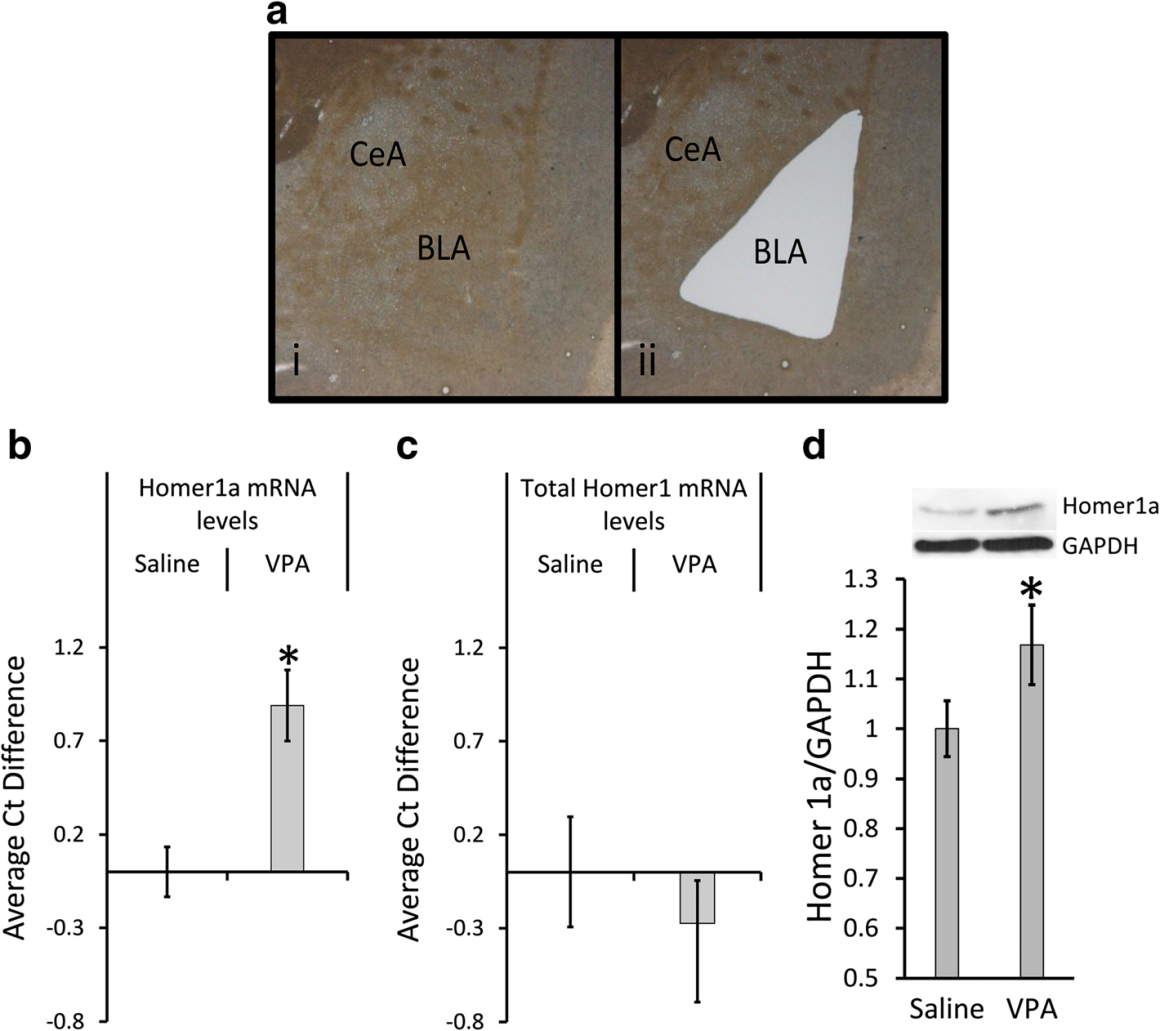
Upregulation of BLA Homer1a mRNA in a different cohort of VPA-exposed animals. **a** Representative picture of before (*i*) and after (*ii*) laser microdissection of the BLA tissue. **b** qRT-PCR for *Homer1a* revealed an upregulation of *Homer1a* mRNA in an independent cohort of VPA-exposed animals compared to saline-exposed animals. **c** qRT-PCR for *Homer1*, which can identify all isoforms of Homer, did not reveal an overall upregulation of *Homer1* in VPA-exposed animals compared to saline animals. **d** Western blot depicting upregulation of Homer1a (~27 kDa) in VPA-exposed animals compared to saline controls. *Bars* represent the mean ± standard error of the mean (SEM) (*p < 0.05)

DNA microarray data can be prone to error, due to technical and biological variability, and therefore, there is a risk of identifying genes that are false positives using this technology. Therefore, to circumvent this potential issue, we examined Homer1a expression in an independent cohort of animals exposed to VPA or saline. Adult animals that had been exposed to VPA or saline, in utero, were sacrificed, the BLA was laser microdissected, and qRT-PCR was performed on RNA extracted from this tissue (Fig. 2a). Significant upregulation of *Homer1a* mRNA was observed as revealed by the average Ct difference of 0.88 ± 0.19 which corresponds to 1.85-fold increase [*t*_(18)_ = 4.09, *p* = 0.006, saline = 10, VPA = 10] in *Homer1a* mRNA levels in VPA animals compared to saline animals (Fig. 2b). *Homer1a* is produced by the conversion of an intronic to exonic sequence in a transcript that normally would code for the long forms of Homer1 [75]. Therefore, we next examined if total levels of Homer1 transcripts (i.e., all isoforms of Homer1) were dysregulated within the BLA due to VPA exposure. To do this, we repeated the qRT-PCR, with PCR primers that detect all Homer1 transcripts. However, there was no difference in the total *Homer1* mRNA levels [*t*_(14)_ = −0.69, *p* = 0.49, saline = 9, VPA = 7] in VPA-exposed animals compared to saline animals (Fig. 2c). Next, we examined if Homer1a protein levels were increased within the BLA following VPA exposure. Adult VPA- and saline-exposed animals were sacrificed, the BLA was microdissected, and protein from this tissue was examined by western blot. This experiment revealed a significant upregulation of Homer1a protein [*t*_(30)_ = −2.26, *p* = 0.04, saline = 16, VPA = 16] in the BLA of VPA compared to saline-exposed animals (Fig. 2d). Collectively, these data indicate that *Homer1a* is upregulated at both mRNA and protein levels across various cohorts of VPA-exposed animals.

### Viral-mediated overexpression of Homer1a in the BLA impaired social interaction but did not affect innate fear and locomotory behavior

**Fig. 3:**
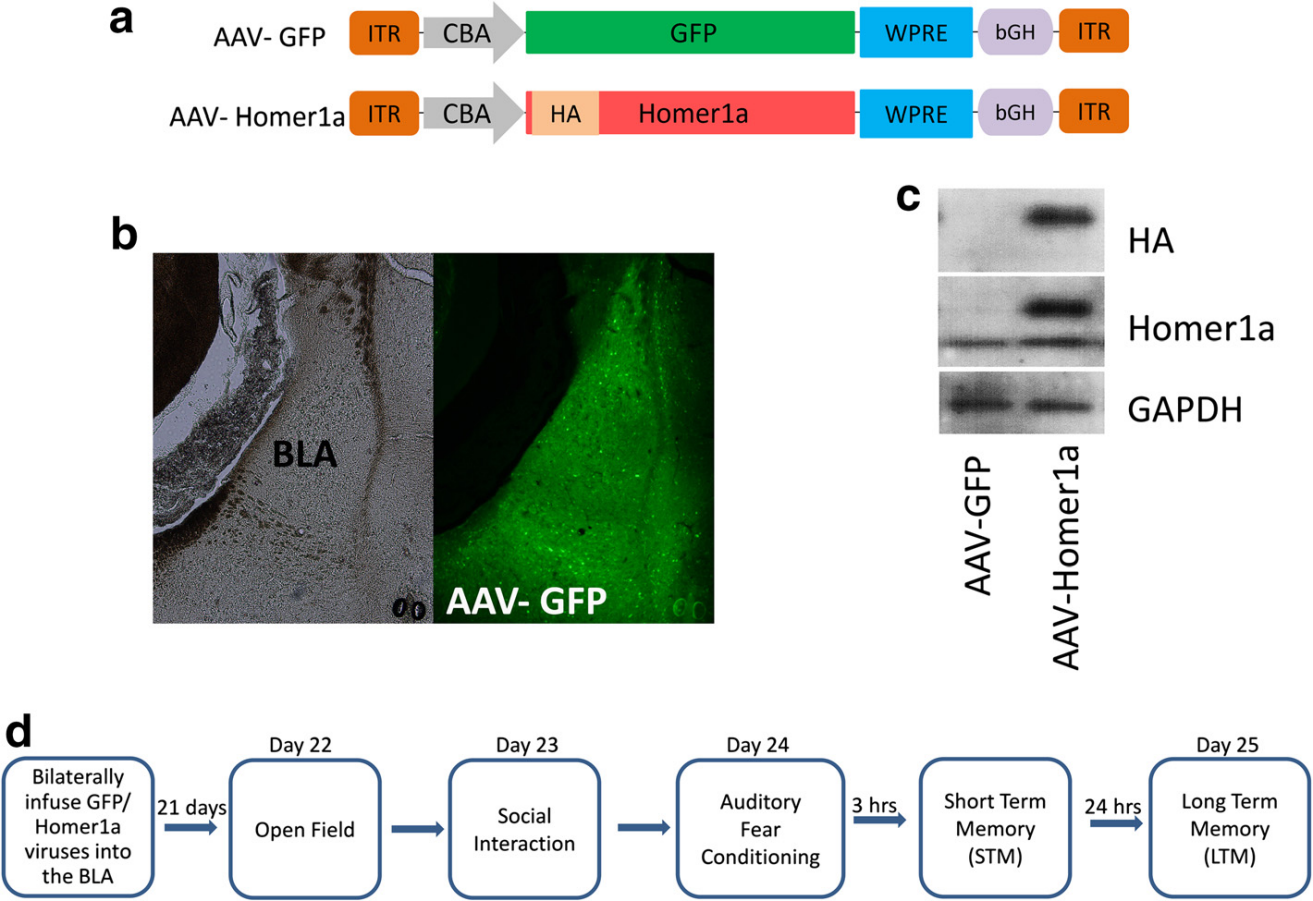
Validation that AAV viruses harboring Homer1a or GFP transgenes function as intended. **a** Viral genome maps for viruses designed to express Homer1a and GFP. **b** Representative picture showing GFP infection in the BLA of a naïve animal. **c** Western blot picture depicting HA tagged Homer1a transgene expression at ~30 kDa which is absent in the GFP animals. A separate band at ~27 kDa shows endogenous Homer1a which is present in both Homer1a and GFP virus-infused groups as expected. **d** A timeline showing the sequence of surgeries and experiments

**Fig. 4:**
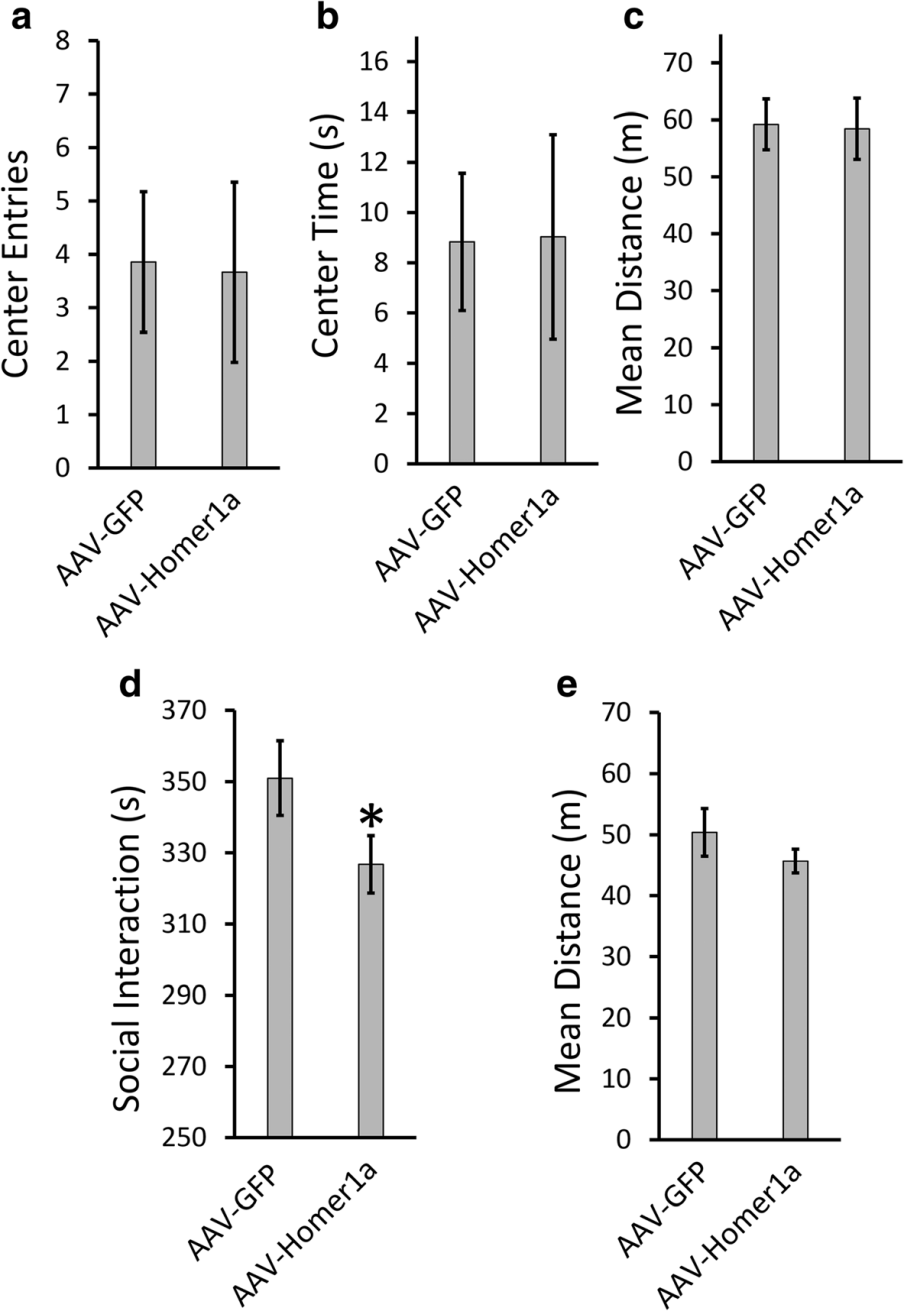
Viral mediated overexpression of Homer1a in the BLA impaired social interaction but did not influence innate fear and locomotory behavior: Animals overexpressing Homer1a exhibit anxiety levels similar to GFP animals in the open field as indicated by (**a**) a similar number of center entries and (**b**) similar center time as compared to GFP animals. **c** The mean total distance traveled was not different between the groups (GFP = 8, Homer1a = 9). Animals overexpressing Homer1a exhibit reduced social interaction compared to GFP animals as indicated by (**d**) reduced social interaction time without affecting (**e**) mean distance traveled in the social interaction apparatus. *Bars* represent the mean ± standard error of the mean (SEM) (*p < 0.05)

Disrupted social behaviors and increased innate fear are prominent symptoms of a number of psychiatric disorders including autism. We and others have shown that animals exposed to VPA *in utero* have higher levels of anxiety and reduced social interaction, similar to symptoms observed in ASD. Since the BLA contributes an important role in modulating innate fear [13, 76], as well as social behavior [60, 77], we wanted to determine if increased expression of Homer1a within BLA neurons can induce autistic-like symptoms such as impaired social interaction and increased innate fear. AAV viruses designed to express an HA-tagged *Homer1a* transgene or *GFP* transgene (Fig. 3a) were stereotaxically injected into the BLA of naïve animals. Twenty-one days post infusion, the animals were sacrificed, the BLA was microdissected, and total protein homogenates were examined via western blotting to confirm the presence of the HA-tagged Homer1a. The HA-tagged Homer1a was visible as a band slightly larger than the endogenous Homer1a (Fig. 3c). A similar sized band was not detected in the samples that received the GFP virus, as expected. GFP expression of virus was confirmed by examining tissue slices that contained BLA transduced with the AAV-GFP virus using fluorescence microscopy (Fig. 3b). Next, another set of animals were bilaterally infused into the BLA with AAV-Homer1a and AAV-GFP viruses, and 21 days later, the animals underwent a battery of behavioral tests (Fig. 3d). First, the animals were examined in an open field to measure potential changes in locomotor behavior and innate fear. Mann-Whitney *U* test revealed no significant difference between the Homer1a and GFP animals in the number of center entries (*p* = 0.66) and center time spent (*p* = 0.66) (Fig. 4a, b) suggesting overexpression of Homer1a in the BLA did not influence innate fear in this open field task. Additionally, the mean distance traveled (*p* = 0.99) (GFP = 7, Homer1a = 6) by the Homer1a and GFP animals in the open field apparatus were similar (Fig. 4c) indicating that overall locomotory behavior remains unaffected by overexpression of Homer1a in the BLA.

Next, we examined how overexpression of Homer1a within the BLA influences social interaction behavior in another group of animals. Animals received AAV-Homer1a or AAV-GFP as described above and 21 days following viral infusion; the social interaction behavior of these animals was examined within a social interaction apparatus with a novel test rat. We observed that the amount of time spent by the AAV-Homer1a animals with the novel rat was significantly less compared to the amount of time the AAV-GFP animals spent with the novel rat (*p* = 0.04; Mann-Whitney *U* test) (GFP = 8, Homer1a = 9) (Fig. 4d). The Homer1a and GFP animals exhibited similar mean distance traveled values, which indicated that within the social interaction apparatus, these groups did not exhibit differences in locomotor behavior (*p* = 0.12) (Fig. 4e).

### Viral-mediated overexpression of Homer1a impairs auditory fear conditioning

**Fig. 5:**
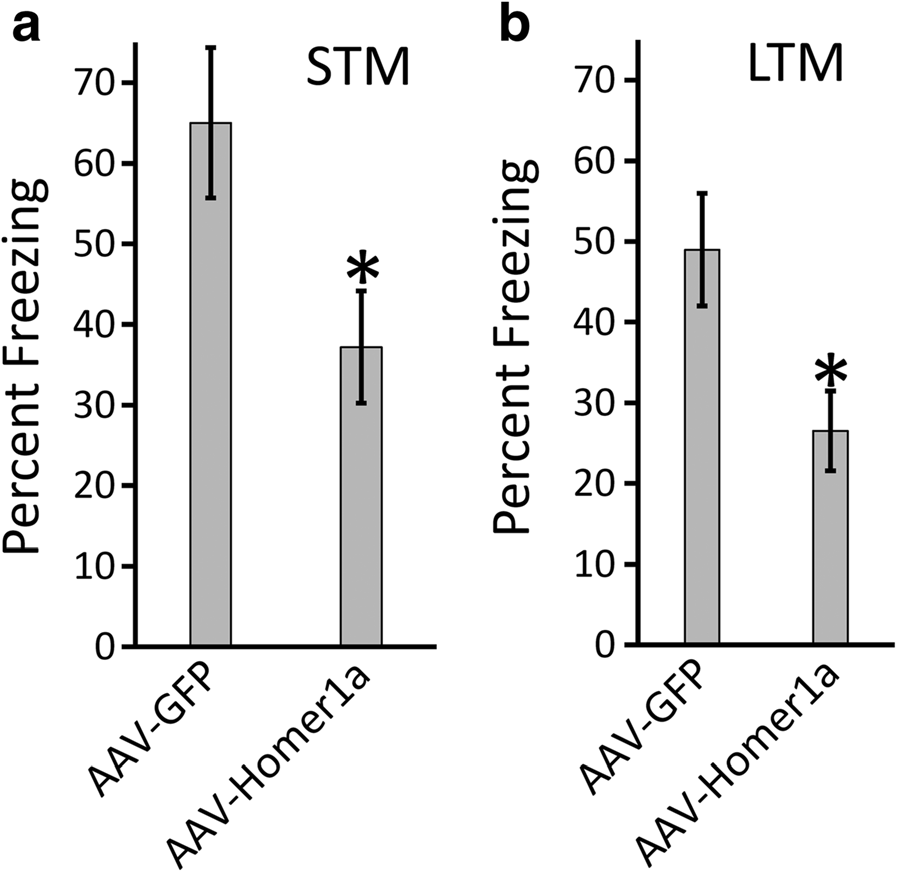
Viral-mediated overexpression of Homer1a in the BLA impaired fear conditioning: Animals infused with either AAV-Homer1a or AAV-GFP were auditory fear conditioned (AAV-GFP = 6, AAV-Homer1a = 6). **a** Auditory fear memory assessed 3 h post fear conditioning (i.e., STM) demonstrated significantly reduced freezing in AAV-Homer1a animals. **b** Auditory fear memory assessed 24 h post fear conditioning (i.e., LTM) demonstrated significantly reduced freezing in AAV Homer1a animals compared to GFP animals. *Bars* represent the mean ± standard error of the mean (SEM) (*p < 0.05)

To determine if overexpression of Homer1a in the BLA impaired auditory fear conditioning, animals were infused with AAV-Homer1a and AAV-GFP as described above, and 21 days later, the animals were subjected to auditory fear conditioning, which consisted of a 30-s, 5-kHz, 75-dB tone that co-terminated with a 0.75-mA foot shock. Animals across the two groups appeared to experience the foot shocks to the same degree as indicated by their response to the shock (i.e., jumping, flinching, etc.; data not shown). Three hours following fear conditioning training, retention of STM was assessed by exposing the rats to two 5-kHz, 75-dB tones within a novel context and subsequently determining the freezing behavior of each rat, to each tone. The Homer1a animals exhibited significantly reduced freezing behavior compared to the GFP control group as revealed by the Mann-Whitney *U* test (*p* = 0.03) without significant differences in freezing per tone within each group, as revealed by the Friedman test [χ^2^ = 5.39, *p* = 0.07] (Fig. 5a). Twenty-four hours following fear conditioning training, LTM was assessed in a similar manner as STM. The Mann-Whitney *U* test indicated significantly higher freezing in the Homer1a group (*p* = 0.03) compared to the GFP group without significant differences in freezing per tone within each group, as revealed by the Friedman test [χ^2^ = 5.78, *p* = 0.67] (GFP = 6, Homer1a = 6) (Fig. 5b). Considering that all animals reacted strongly to receiving the foot shock during fear conditioning, the differences in freezing between the groups during STM and LTM, is likely not due to differences in pain perception but rather due to differences in learning.

**Fig. 6:**
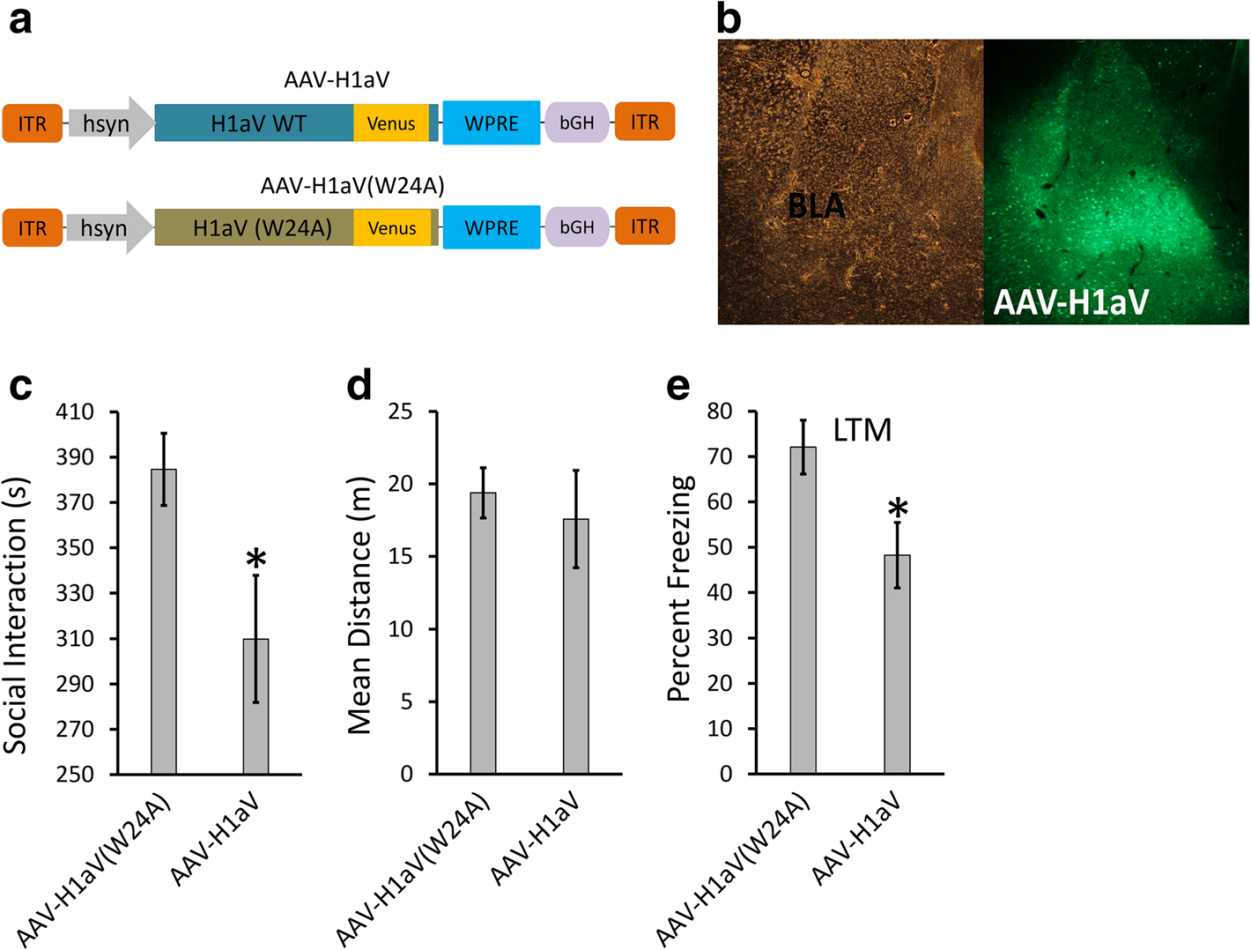
Viral-mediated overexpression of AAV-H1aV in the BLA impaired social interaction and fear conditioning: Animals were infused with either AAV-H1aV designed to overexpress Homer1a or AAV-H1aV(W24A) designed to expressed a mutant form of Homer1a, which served as a control. **a** Viral genome maps depicted for AAV-H1aV and AAV-H1aV(W24A). **b** Representative picture depicting AAV-H1aV viral transduction in the BLA of naïve animals. AAV-H1aV animals exhibit reduced social interaction compared to AAV-H1aV(W24A) as indicated by (**c**) reduced social interaction time without affecting (**d**) mean distance traveled in the social interaction apparatus (AAV-H1aV(W24A) = 7, AAV-H1aV = 8). **e** Animals expressing AAV-H1aV and AAV-H1aV(W24A) were subjected to auditory fear conditioning, and 24 h later, they were examined for LTM. AAV-H1aV animals exhibited significantly reduced freezing compared to AAV-H1aV(W24A) animals

In our final set of experiments, we wanted to reproduce the main effects we observed in the social interaction and fear conditioning tests when Homer1a was overexpressed in BLA neurons. However, this time we used AAV viruses that were designed to express Homer1a with Venus fluorescent protein as a c-terminal fusion (H1aV). The control virus was similarly designed but contained a point mutation (W24A) in the EVH1 domain of H1aV, which abolishes the ability of Homer1a to interact with postsynaptic protein partners (H1aV(W24A)) (Fig. 6a). The animals were infused with either H1aV or H1aV(W24A) (Fig. 6b), and twenty-one days later, they were subjected to social interaction testing and auditory fear conditioning as described above. During social interaction, we observed that H1aV animals spent significantly less time with a novel test rat compared to H1aV(W24A) (*p* = 0.04) as revealed by Mann-Whitney *U* test while the mean distance traveled in the social interaction apparatus was similar between both groups (*p* = 0.09) (Fig. 6c, d). Next, the animals were subjected to auditory fear conditioning, which consisted of a 30-s, 5-kHz, 75-dB tone that co-terminated with a 0.75-mA foot shock. Animals across the two groups appeared to experience the foot shocks to the same degree as indicated by their response to the shock (i.e., jumping, flinching, etc.; data not shown). Twenty-four hours following fear conditioning, the H1aV animals exhibited significantly reduced freezing compared to animals that received H1aV(W24A) [*p* = 0.02; Mann-Whitney *U* test, AAV-H1aV(W24A) = 7, AAV-H1aV = 9] (Fig. 6e) without significant differences in freezing per tone within each group, as revealed by the Friedman test [*χ*^2^ = 1.238, *p* = 0.53]. Collectively, these data indicate that overexpression of Homer1a in the BLA impairs social interaction and fear conditioning compared to the H1aV(W24A) control animals.

## Discussion

A growing body of literature has identified a range of autism-like behavioral and synaptic abnormalities in animals exposed to VPA in utero. This includes a previous study from our lab where we identified fear learning and social abnormalities in VPA-exposed animals. In this study, we identified *Homer1a* as one of the molecules that was significantly upregulated in the BLA of VPA-exposed animals at both the mRNA and protein levels. We hypothesized that upregulation of Homer1a in the BLA might be contributing to the behavioral abnormalities observed in VPA-exposed animals. To test our hypothesis, we used a viral mediated approach to overexpress Homer1a in the BLA of naïve animals. Our results revealed for the first time that overexpression of Homer1a within BLA neurons was capable of impairing auditory fear conditioning and social interaction. These results are very interesting in part because we were able to demonstrate a possible causal mechanism underlying fear conditioning and social interaction abnormalities observed in an animal model of autism.

Homer is a family of scaffolding proteins found at the postsynaptic density. Homer1b/c is a longer isoform of the family which interacts with a number of proteins at the postsynaptic density. It has an EVH1 domain and a proline-rich containing motif at the N-terminus, which binds to various scaffolding and signal transduction molecules, such as type I mGluR, IP3 receptors (IP3R), Shank, transient receptor potential canonical (TRPC) family channels, and dynamin3 [49, 78–81]. The C-terminus of long isoforms of Homer1 contain a coiled-coil domain followed by two leucine zipper motifs which promotes homomeric or heteromeric interactions with other long isoforms of Homer to form a network and stabilize the integrity of the postsynaptic density [50]. *Homer1a* is an immediate early gene (IEG) that codes for a shorter isoform of Homer1, which lacks the coiled-coil domain and the ability to interact with other Homer1 molecules. This allows Homer1a to act as a dominant negative regulator by disrupting the interaction of the long isoforms and therefore allows Homer1a to reorganize the postsynaptic density. Homer1a is also involved in intracellular calcium homeostasis, receptor trafficking, gene transcription, signal transduction, and homeostatic synaptic downscaling [48, 57, 82, 83]. Homer1a also plays a role in pain plasticity by protecting against chronic inflammatory pain without affecting the basal pain threshold [76]. Additionally, Homer1a contributes an important role in learning and memory, and *Homer1a* knockout animals exhibit reduced fear memory acquisition as well as reduced short-term memory [56]. Overexpression of Homer1a inhibits dendritic spine morphogenesis as well as reduces the size of PSD95 clusters, *N*-methyl-d-aspartate (NMDA) receptor clusters and surface levels of α-amino-3-hydroxy-5-methyl-4-isoxazolepropionic acid (AMPA) receptors [84]. These findings indicate that either a deficit or excess of Homer1a could be pathological and prevent normal synaptic functioning.

There is evidence indicating that dysregulation of Homer1 may be occurring in a sub-population of ASD patients. For example, a study comprised of a population diagnosed with non-syndromic autism identified a significant number of genes involved in the mGluR5 pathway including Homer1 [64]. Specifically, single nucleotide variants (SNV) were identified, which localized to the EVH1 domain and 3′ untranslated region (UTR) region of Homer1. A separate postmortem study found reduced Homer1 protein expression in the frontal cortex of ASD patients [85]. Moreover, different animal models of autism have indicated that Homer1 and Homer1a function may be dysregulated in these models. One study found Homer1a to have an increased interaction to mGluR5 receptors in the hippocampus, which would disrupt Homer1 scaffolds at the synapse of fragile X mice. Increased interaction of Homer1a to mGluR5 in turn enhanced mGluR5-dependent long-term depression (LTD) [38]. Interestingly, a different study found an increased interaction of mGluR5 to the long isoform of Homer1, and it was found that this increased interaction also enhanced mGluR5-dependent LTD in an Angelman syndrome mouse model [86]. Surprisingly, in the Angelman Syndrome study, changes in the coupling of mGluR5 receptors to Homer proteins were opposite to those seen in fragile X. Nevertheless, these studies underscore the significant role of Homer1a in the neurobiological mechanisms leading to ASD.

Our microarray data also revealed other genes such as *Mef2C*, *gfap*, *grxcr1*, and *sgk1*, which were dysregulated in VPA-exposed animals (complete gene list in Additional file 1.). Homer1a’s previously established role in learning and its association with human ASD made it an attractive candidate to pursue in the present study. However, it will be interesting to investigate the role of these other genes in the future to determine how they may contribute to the aberrant behavioral and synaptic abnormalities seen in the VPA-exposed animals. We propose that dysregulation of many genes, which include *Homer1a*, gives rise to the ASD phenotype seen in VPA-exposed animals, and it would be unlikely that Homer1a alone is solely impairing fear conditioning or social interaction. Our experimental data clearly indicate that Homer1a is consistently upregulated in neurons from the BLA of VPA-exposed animals and that when we overexpress Homer1a within the BLA utilizing viral mediated gene delivery, we observe impairments in fear conditioning and social interaction behavior compared to the control groups. However, an important caveat of these viral-mediated gene delivery experiments is that they do not provide the ability to precisely adjust Homer1a levels to mirror what is occurring exactly within VPA-exposed animals.

It is well known that the amygdala can modulate social behavior and is a hub for brain networks that support social life. For example, bilateral lesions of the amygdala in monkeys can reduce social behavior and increase social phobia [77] and neonatal amygdala lesions suppressed social interactions in adult rats [87]. A recent study demonstrated a critical role for BLA neurons that project to the hippocampus in bi-directionally modulating social behavior. This study demonstrated that over activation of these BLA projection neurons could reduce social interaction [88]. However, the molecular mechanisms in the amygdala that influence social behavior still remain largely unknown. For the first time, we demonstrate that overexpression of Homer1a in BLA neurons impairs social interaction in naïve animals.

Valproic acid is a histone deacetylase (HDAC) inhibitor and is prescribed for the treatment of epilepsy [89–91]. The mechanism by which VPA acts at the molecular level is currently not fully understood. Pups exposed to VPA *in utero* exhibited increased total brain, brain-derived neurotrophic factor (BDNF) expression and abnormal development of serotonergic neurons in the dorsal raphe nucleus [92, 93]. Various biochemical studies indicate that VPA can suppress neuronal activity by blocking sodium and calcium channels and enhance the functioning of the inhibitory neurotransmitter, gamma-aminobutyric acid (GABA), in the brain [94, 95]. Further, in an attempt to understand the mechanism of VPA-induced toxicity, several studies found dysregulated gene expression related to organ morphogenesis and neural tube defects [96–100], and these affects have been attributed to VPA’s ability to inhibit histone deacetylase [101–103]. Since HDAC plays an important role in regulating transcription during fetal development [45, 104, 105], it is possible that VPA may induce abnormal gene expression during embryogenesis, causing autism-like behavioral impairments. For example, a recent rodent study demonstrated that inhibition of HDAC *in utero* is sufficient to cause autism-like phenotypes including sociability deficits in exposed offspring [106]. Further research needs to be conducted to understand how exposure to VPA *in utero* is causing overexpression of Homer1a.

## Conclusions

ASD is a heterogeneous disorder with a complex underlying molecular mechanism. It is very likely that the behavioral impairments observed in an ASD individual are caused by multiple synaptic deficits. However, Homer1a contributes a significant role in synaptic plasticity within a larger glutamatergic system—dysregulation of which has been clearly implicated in ASD [64, 107–109]. Our findings indicate that overexpression of Homer1a in the BLA can induce autism-like symptoms which provides novel and interesting information for the ASD research community and can be further investigated for their role as a possible therapeutic intervention through genetic or pharmacological manipulations.

## Additional files

Additional file 1:
**Table comparing parametric and non-parametric statistical outcome for selected** ***behavioral experiments.*** For behavioral experiments with sample sizes of *n* < 10 per group, statistical analyses were performed using both parametric and non-parametric statistical analysis to eliminate chances of normality assumption violation. (XLSX 15 kb) Additional file 2:
**Table of microarray data from VPA exposed rats compared to saline exposed control** ***animals*** **.** Gene lists were created based on the criteria that the gene must exhibit an average fold difference of 1.5 fold or greater in pair wise comparisons between VPA and saline amygdala samples with a *t*-test p-value of *p* < 0.05. Microarray data listed, with Probe ID, Gene symbol, Gene name, etc. (XLSX 16 kb)

## Abbreviations

AAV: adeno-associated virus
ADHD: attention deficit hyperactivity disorder
AMPA: α-amino-3-hydroxy-5-methyl-4-isoxazolepropionic acid
aRNA: amplified RNA
ASD: autism spectrum disorder
BDNF: brain-derived neurotrophic factor
E: embryonic
GABA: gamma-aminobutyric acid
GAPDH: glyceraldehyde-3-phosphate dehydrogenase
HDAC: histone deacetylase
IEG: immediate early gene
IP3R: IP3 receptors
LMD: laser microdissection
LTD: long-term depression
LTM: long-term memory
mGluR: metabotropic glutamate receptors
NMDA: *N*-methyl-d-aspartate
PD: postnatal day
qRT-PCR: quantitative PCR
SNV: single nucleotide variants
STM: short-term memory
TRPC: transient receptor potential canonical
UTR: untranslated region
VPA: valproic acid
